# Antibody Kinetics of Immunological Memory in SARS-CoV-2-Vaccinated Healthcare Workers—The ORCHESTRA Project

**DOI:** 10.3390/vaccines13060611

**Published:** 2025-06-05

**Authors:** Seyedalireza Seyedi, Sara Sottile, Mahsa Abedini, Paolo Boffetta, Francesco Saverio Violante, Vittorio Lodi, Giuseppe De Palma, Emma Sala, Marcella Mauro, Francesca Rui, Stefano Porru, Gianluca Spiteri, Luigi Vimercati, Luigi De Maria, Pere Toran-Monserrat, Concepción Violán, Eleonóra Fabiánová, Jana Oravec Bérešová, Violeta Calota, Andra Neamtu

**Affiliations:** 1 Department of Economics, University of Bologna, 40126 Bologna, Italy; seyedalireza.seyedi2@unibo.it; 2 Department of Medical and Surgical Sciences, University of Bologna, 40126 Bologna, Italy; sara.sottile4@unibo.it (S.S.); mahsa.abedini@unibo.it (M.A.); francesco.violante@unibo.it (F.S.V.); vittorio.lodi@aosp.bo.it (V.L.); 3 Stony Brook Cancer Center, Stony Brook University, Stony Brook, NY 11794, USA; 4 IRCCS Azienda Ospedaliero-Universitaria di Bologna—Policlinico di Sant’Orsola, 40138 Bologna, Italy; 5 Department of Medical and Surgical Specialties, Radiological Sciences and Public Health, Unit of Occupational Health and Industrial Hygiene, University of Brescia, 25121 Brescia, Italy; giuseppe.depalma@unibs.it (G.D.P.); emma.sala@unibs.it (E.S.); 6 Unit of Occupational Medicine, University of Trieste, 34127 Trieste, Italy; mmauro@units.it (M.M.); frui@units.it (F.R.); 7 Section of Occupational Medicine, Department of Diagnostics and Public Health, University of Verona, 37134 Verona, Italy; stefano.porru@univr.it; 8 Occupational Medicine Unit, University Hospital of Verona, 37134 Verona, Italy; gianluca.spiteri@aovr.veneto.it; 9 Interdisciplinary Department of Medicine, University of Bari, 70121 Bari, Italy; luigi.vimercati@uniba.it (L.V.); luigi.demaria@uniba.it (L.D.M.); 10 Unitat de Suport a la Recerca Metropolitana Nord, Institut Universitari d’Investigació en Atenció Primària Jordi Gol (IDIAP Jordi Gol), Mare de Déu de Guadalupe, 2, 08303 Mataró, Spain; pere.toran@udg.edu (P.T.-M.); cviolanf.mn.ics@gencat.cat (C.V.); 11 Germans Trias i Pujol Research Institute (IGTP), Ctra de Can Ruti, Camí de les Escoles s/n Street, 08916 Badalona, Spain; 12 Department of Medicine, Faculty of Medicine, Universitat de Girona, Emili Grahit, 77, 17071 Girona, Spain; 13 Multidisciplinary Research Group in Health and Society (GREMSAS) (2021-SGR-0148), Institut Universitari d’Investigació en Atenció Primària Jordi Gol (IDIAPJGol), Mare de Déu de Guadalupe, 2, 08303 Barcelona, Spain; 14 Grup de REcerca en Impacte de les Malalties Cròniques i les seves Trajectòries (GRIMTra) (2021 SGR 01537), Institut Universitari d’Investigació en Atenció Primària Jordi Gol (IDIAPJGol), Mare de Déu de Guadalupe, 2, 08303 Barcelona, Spain; 15 Immunology Department, FOCIS Center of Excellence, Universitat Autnoma de Barcelona, Plaça Cívica, 1, 08913 Cerdanyola del Vallès, Spain; 16 Immunology Division, Laboratori Clinic Metropolitana Nord (LCMN), Hospital Universitari Germans Trias i Pujol, Carretera del Canyet s/n, 08916 Badalona, Spain; 17 Department of Medicine, Universitat Autònoma de Barcelona, PlaÇa Civica, 1, 08193 Cerdanyola de Vallès, Spain; 18 Red de Investigación en Cronicidad, Atención Primaria y Prevención y Promoción de la Salut (RICAPPS), Instituto de Salud Carlos III (ISCIII), Avenida Monforte de Lemos, 5, 28029 Madrid, Spain; 19 Occupational Health Department, Regional Authority of Public Health, 97556 Banská Bystrica, Slovakia; eleonora.fabianova@vzbb.sk; 20 Faculty of Health, Catholic University, 03401 Ružomberok, Slovakia; 21 Epidemiology Department, Regional Authority of Public Health, 97556 Banská Bystrica, Slovakia; beresova@vzbb.sk; 22 National Institute of Public Health, 050463 Bucharest, Romania; violeta.calota@insp.gov.ro (V.C.); andra.neamtu@insp.gov.ro (A.N.)

**Keywords:** SARS-CoV-2, vaccine, serology, antibody, immunization, healthcare workers, bass diffusion

## Abstract

**Background/Objectives:** This study examines the longitudinal dynamics of anti-nucleocapsid (anti-N) and anti-spike (anti-S) antibody responses to SARS-CoV-2 infection and mRNA vaccination based on 81,878 serum samples from 23,616 healthcare workers (HCWs) across five European countries. It includes data across four scheduled vaccine doses—predominantly BNT162b2—with 25% of samples originating from individuals with confirmed prior infection, as evidenced by elevated anti-S levels, positive Anti-N antibodies, or PCR results. **Methods:** The study employed a shifted transformation method for data normalization and utilized the Bass diffusion model to predict antibody titer dynamics influenced by both internal factors—such as immune activation contextualized through sociodemographic issues—and external factors, including infection and vaccination. Despite the absence of direct measurements for some internal variables, the model effectively inferred their impact, enabling a rigorous and nuanced delineation of immune response profiles. **Results:** The Bass diffusion model rigorously captured variations in antibody titers, analyzed through demographic factors such as gender, age, and job role, while thoroughly accounting for pre-infection status. The results indicate that Anti-N antibodies, exclusively produced post-infection, exhibited a rapid decline, while anti-S antibodies, generated from both infection and vaccination, demonstrated prolonged persistence. A significant decline in anti-S levels was observed 3–5 months post-vaccination, with adaptive immunity—characterized by the dominance of internal factors effects relative to external ones—achieved in most groups after the fourth dose. However, adaptive immunity post second dose was limited to specific demographics. **Conclusions:** These findings emphasize the significance of the Bass Method in predicting vaccine-induced, hybrid immune responses and detecting adaptive immunity by overcoming limitations in internal factor data, thereby advancing effective vaccination and infection control strategies during public health crises. These findings highlight the Bass Method’s value in predicting vaccine-induced and hybrid immunity, effectively addressing internal factor data gaps to enhance vaccination and infection control strategies.

## 1. Introduction

Understanding antibody kinetics is crucial for evaluating immune responses to SARS-CoV-2 infection and COVID-19 vaccination. The primary immune response following initial infection or vaccination is characterized by a lag phase, succeeded by a rapid increase in IgM and IgG antibodies, which subsequently decline as the antigen is cleared [1]. A repeated factor, whether through natural infection or booster vaccinations, elicits a more robust secondary response mediated by memory B cells, resulting in the rapid production of high-affinity IgG antibodies [2]. This differentiation is crucial in the context of COVID-19, as Anti-N and anti-S antibodies serve distinct functions. Anti-N antibodies, which target the nucleocapsid protein, are produced shortly after SARS-CoV-2 infection and decline within a few months, rendering them reliable markers of recent infection [3]. In contrast, anti-S antibodies, which target the spike protein, are produced following both infection and vaccination, with vaccination generally yielding higher titers [4,5]. These antibodies peak shortly after vaccination and can persist for up to 12 months, indicating sustained immunity and vaccine effectiveness [6]. Empirical data, including studies on the mRNA vaccine, demonstrate sustained IgG responses over several months, thereby supporting long-term vaccine effectiveness [7,8]. Specifically, the concurrent monitoring of Anti-N and anti-S antibodies enables differentiation between natural infection and vaccination, while providing critical insight into the persistence of vaccine-induced immunity [9,10]. A key challenge in this monitoring effort is addressing gaps in antibody data during critical periods, particularly when novel viruses emerge and there is limited information regarding their immunological responses and the role of their responders, such as immune cell activation. The use of mathematical methods, especially predictive diffusion models that have proven effective in finance, pharmaceutical sciences, and medicine, offers a promising strategy to address this issue. Therefore, we propose employing the Bass diffusion model to effectively tackle this challenge in SARS-CoV-2 cases during the initial stages of vaccine implementation. Originally designed to forecast market adoption of new products [11], this model can be modified to characterize the spread of immune responses in vaccinated populations exposed to emerging pathogens. The model highlights hybrid immunity, where vaccine-induced and adaptive immunity develop simultaneously. This is crucial for early responders acting as “early adopters” of a novel vaccine for a newly identified viral infection. Bass diffusion modeling quantifies antibody production and decay by integrating internal factors (immune activation within a sociodemographic context) and external factors (infection, vaccination), providing a robust statistical framework to predict the performance and persistence of the immune response over time. This approach offers a superior alternative to traditional regression models, which are often limited by noisy data and lack the ability to simultaneously capture antibody fluctuations and the influences of their underlying drivers. By innovatively forecasting the peak and duration of antibody levels post-infection and vaccination, it significantly enhances public health strategies and vaccine rollout plans. This article employs Bass diffusion modeling to predict antibody kinetics against COVID-19, elucidating immune response dynamics amid data complexity, using Anti-N and anti-S profiles and PCR testing to identify prior infection from 81,878 serum samples of 23,616 HCWs across five European countries following infection and up to four mRNA vaccine doses. By integrating empirical antibody data, the study provides critical insights into vaccine effectiveness, immune response patterns, and protection durability. These findings are crucial for optimizing vaccination strategies and enhancing public health outcomes, as they offer a data-driven framework for assessing vaccine effectiveness, distinguishing hybrid immunity, and monitoring adaptive immune memory.

## 2. Materials and Methods

### 2.1. Study Design

The ORCHESTRA study (https://orchestra-cohort.eu/ (accessed on 29 May 2025)) is a large, multicenter, prospective cohort involving over 80,000 healthcare workers (HCWs) from European hospitals and primary health centers. The study commenced in December 2020 and continued until November 2024, with the objective of assessing serological outcomes 27 months following the initial vaccination [12,13]. The study includes HCWs from Germany (Munich), Italy (seven centers), Spain (two centers), and multiple centers in Slovakia and Romania [14,15,16]. Due to insufficient data, three centers (Padua, Perugia (Italy), Oviedo (Spain)) were excluded. A total of 23,616 HCWs were analysed, resulting in 81,878 serological measurements, 31,570 Anti-N assays, and 17,333 PCR tests. The primary outcome measured was anti-S antibody levels, obtained from medical records and standardized testing across centers [17]. Data were normalized using trigonometric and log10 transformations [18]. To predict adaptive immunity in the absence of data on immune cell activation, we utilized the Bass diffusion model. This model serves not only as a critical tool for predicting parameters without a known history, such as antibody kinetics in emerging diseases like COVID-19, but also for assessing the extent to which internal factors, including immune cell activation and influence antibody levels, even when the magnitude of these factors is unknown. To assess the robustness of the model over time, we incorporated various factors, including vaccination timing, prior infection, gender, age, and job title. Data on job titles were available for 97% of cases, with minor inconsistencies that did not significantly affect the analysis. The study employed cohort-specific serological assays to quantify post-vaccination SARS-CoV-2–specific antibody responses, focusing on anti-spike antibody titers as detailed in Supplemental Table S1. Assay platforms varied across sites and included Roche Elecsys^®^, Abbott IgG II Quant, as well as other ELISA- and CLIA-based methods [12,13,14,15,16,19]. To ensure cross-cohort comparability, all antibody measurements were harmonized to WHO binding antibody units per milliliter (BAU/mL) using validated conversion factors. The study population was categorized as “pre-infected” or “non-pre-infected” based on prior SARS-CoV-2 status. Pre-infection was defined by a positive PCR result and/or a positive Anti-N test accompanied by elevated anti-S antibodies, while non-pre-infected individuals tested negative by PCR or anti-N. Despite limitations—such as the waning of Anti-N antibodies and PCR’s restriction to active infection—this combined approach enabled a robust comparison of immune responses, particularly after revaccination. Statistical analyses were conducted using R software version 4.1.1. The study received approval from the Italian Medicines Agency (AIFA), the Ethics Committee of the Italian National Institute of Infectious Diseases (INMI) Lazzaro Spallanzani, and the local ethics committees for each participating group.

### 2.2. Statistical Analysis

**Figure 1:**
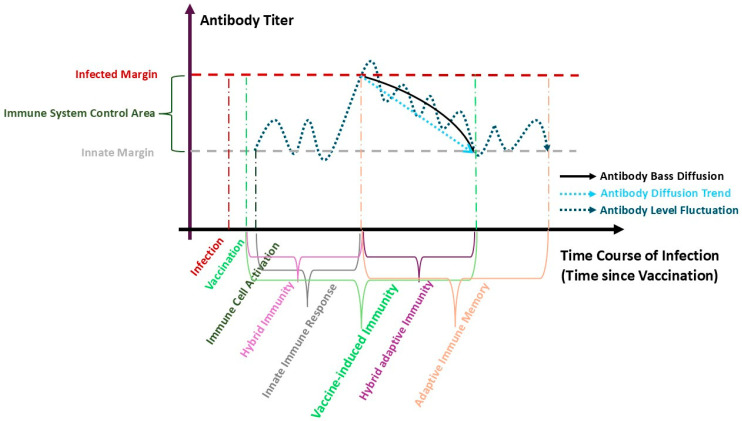
**Antibody Bass Diffusion Model of SARS-CoV-2 Immune Dynamics.** Schematic of SARS-CoV-2 antibody kinetics (*y*-axis) over time since vaccination/infection (*x*-axis), with titers fluctuating between the Infected Margin (dark red dashed line) and Innate Margin (gray dashed line). The Antibody Fluctuation curve (dark blue dashed line) captures cohort-level serological trends, overlaid with the Bass Diffusion Fit (solid black line) modeling overall kinetics. Its decomposition yields the Diffusion Trend (light blue dashed line), reflecting net antibody secretion driven by antigenic exposure and immunological amplification. Below, immunological phases are color-annotated: Infection (dark red), Vaccination (light green), Immune Cell Activation (dark green), Innate Immunity (dark gray), Adaptive Immunity (orange), Hybrid Immunity (purple), Hybrid Adaptive Immunity (dark purple), and Vaccine-Induced Immunity (green). This framework quantifies the interplay of internal (T/S-cell help) and external (vaccination/infection) drivers shaping immunological memory.

Diffusion is a fundamental process across various domains, including physical, biological, and social systems [20]. The Bass model, introduced by Frank Bass in 1969 [11], establishes a relationship between adoption rates and prior adopters. Widely utilized to forecast demand peaks, it aids in production and distribution planning by elucidating the dynamics of innovation diffusion. Its use has been extended to medical technologies, such as pharmaceutical contamination curves and drug adoption [21,22,23]. The model also parallels epidemiological models, particularly in understanding the spread of infection [24,25,26,27,28]. This study elucidates the application of the Bass model in forecasting the propagation of SARS-CoV-2 infections through an analysis of immune responses, with a particular emphasis on fluctuations in antibody secretion (see Figure 1). Our methodology encompasses an in-depth analysis of serological test results from HCWs obtained during the intervals between mRNA vaccine doses. By employing the Bass model, we aspire to generate nuanced insights into hybrid immunity among individuals who have experienced both prior infection and vaccination. Additionally, we investigate the development of adaptive immune memory in both pre-infected and non-pre-infected vaccinated groups, taking into account the effects of immune cell activation, independent of direct data. The primary objective of this analysis is to rigorously assess the duration of protective immunity conferred upon these populations. In greater detail, the proposed model delineates the influence of internal factors, such as immune cell activation, and external factors, including vaccination and infection, on antibody dynamics through a Bass diffusion framework characterized by three parameters: the maximum serological measurement (m) and the coefficients (p) and (q) for external and internal factors, respectively. This differentiation facilitates the assessment of vaccine-induced immunity and enhances predictions regarding the duration and type of immune memory. The model proficiently predicts antibody levels among HCWs between vaccine doses, discerning fluctuations attributable to vaccination and infection while delineating the return to baseline levels, which signifies long-term protection despite the limitations imposed by inadequate factor data. Furthermore, it surpasses traditional regression models by skillfully navigating complex and heterogeneous datasets derived from diverse cohorts [11,29]. Although the prediction procedure may yield low R-squared values, reflecting the complexity of real-world data and providing nuanced insights [30], the Bass model avoids negative R-squared values, ensuring greater stability [31] and making it particularly well-suited for modeling adoption and forecasting diffusion processes [29,32]. The Bass model utilizes the External Dominance Ratio (k=p/q) and the internal dominance ratio (1/k=q/p) to rigorously evaluate the influences of external and internal factors on the adoption of innovative medical products [22]. This analytical framework encompasses vaccines that enhance antibody secretion beyond a defined threshold, as elucidated by the results of our investigation. This study undertakes a comprehensive predictive analysis of the dominant effects of factors and types of immunity across vaccine doses (from Dose 1 to Dose 4), stratified by demographic characteristics and occupational roles, alongside pre-infection status. Utilizing the Bass diffusion model, we predict anti-S and Anti-N antibody levels in the absence of empirical data on T and S cell dynamics. This framework adeptly captures fluctuations in antibody levels and elucidates the mechanisms underlying hybrid immunity in pre-infected vaccinated populations. Furthermore, it discerns the presence of adaptive immunity by assessing whether internal effects surpass external effects post-vaccination, as indicated by the 1/k ratio exceeding the k ratio derived from the model, thereby determining individuals’ states of vaccine-induced immunity.

## 3. Results

### 3.1. Participants Characteristics

This study examined a large cohort of vaccinated HCWs across five European countries, showing that nearly all received an initial dose of mRNA vaccines—primarily BNT162b2 (Pfizer-BioNTech) and mRNA-1273 (Moderna)—with most completing subsequent doses (see Supplemental Figure S1). The dataset incorporated an extensive number of serological measurements, with nearly all participants contributing at least two samples, and a substantial fraction providing more than four samples. In three specific cohorts (Bologna, Brescia, and Slovakia), anti-S antibody results exceeding the assay cut-off were standardized. Serological testing was primarily conducted in Italy, especially Brescia, with moderate contributions from Spain and smaller amounts from Slovakia, Germany, and Romania; PCR testing was similarly led by Italy, while Anti-N testing was overwhelmingly concentrated in Italy, particularly Brescia, with notable input from Spain and Germany (Supplemental Figure S2).

The findings highlight significant regional disparities in testing efforts, particularly for serological and PCR testing, with Anti-N testing concentrated in specific areas. The largest volume of infectious serological samples was reported in Italy, primarily in Brescia and Trieste, with smaller but notable contributions from Verona and Bologna. Spain’s Barcelona also reported a significant number of infections, while smaller figures were observed in Germany-Munich, Slovakia, and Romania. Non-infectious sample contributions were also most pronounced in Italy, dominated by Brescia, Bologna, and Trieste.

**Figure 2:**
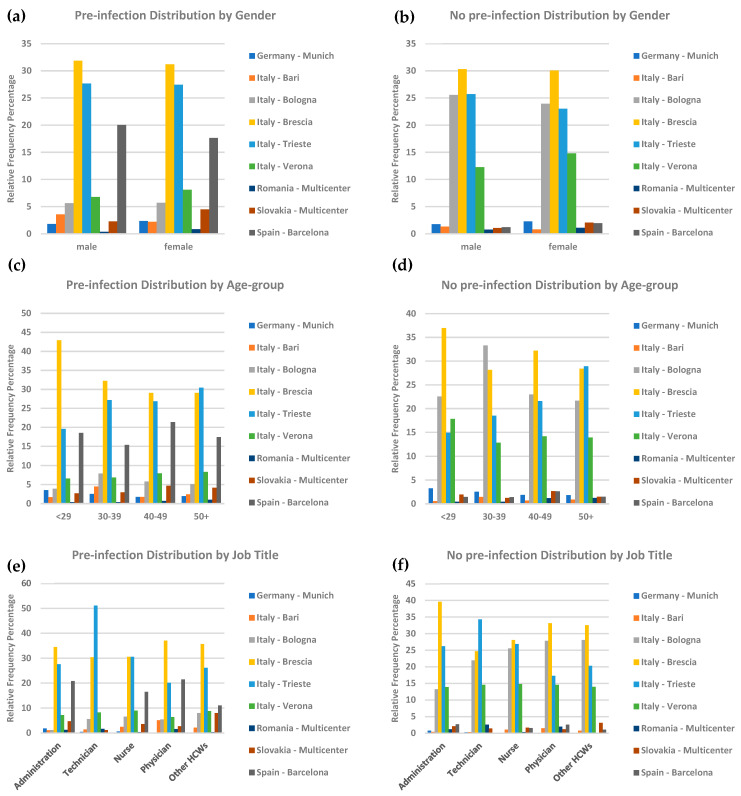
**Population characteristics of SARS-CoV-2 serological measures among vaccinated HCWs across multiple cohorts:** Panels (**a**,**b**) show the frequency distributions of pre-infected and non-pre-infected individuals by gender, highlighting gender-related differences across regions. Panels (**c**,**d**) present the age-group distributions of pre-infected and non-pre-infected individuals, emphasizing trends in infection prevalence across different age cohorts. Panels (**e**,**f**) illustrate occupational patterns, depicting pre-infection and no pre-infection frequencies across various healthcare roles, providing insight into professional disparities. These panels collectively analyze SARS-CoV-2 pre-infection patterns among HCWs from the ORCHESTRA project, with frequencies normalized to account for cohort size variations.

The analysis revealed observable gender- and age-related patterns in the distribution of pre-infected and non-pre-infected individuals across regions (Figure 2a,b). In most regions, women showed a higher proportion of pre-infection cases compared to men, with noticeable variations in Italy-Trieste and Spain-Barcelona (Figure 2a). However, in the group of individuals with no pre-infection, gender distributions appeared more similar in some regions, such as Italy-Brescia (Figure 2b).

Age stratification showed that pre-infection increased with age, with older cohorts (50 years and above) in regions such as Italy-Brescia and Italy-Trieste displaying the highest proportions of pre-infected individuals (Figure 2c). In contrast, younger cohorts, particularly in Germany-Munich, exhibited visibly lower pre-infection rates (Figure 2c). Similarly, individuals with no pre-infection also increased with age, with regions like Italy-Bologna and Italy-Brescia showing the most pronounced patterns in older age groups (Figure 2d). These findings highlight the interplay of demographic factors, particularly gender and age, in shaping serological profiles across diverse geographical regions.

The analysis by job title (Figure 2e,f) revealed notable disparities in pre-infection and no-pre-infection prevalence across regions and professional roles. Nurses and physicians exhibited the highest relative frequencies of pre-infection, particularly within Italian cohorts. Regions such as Italy-Brescia and Italy-Trieste experienced a disproportionately high burden of pre-infection among nurses, with physicians and technicians also making significant contributions to the pre-infected group. Conversely, in regions like Germany-Munich, pre-infection rates were minimal, with negligible contributions from nursing and administrative roles. The distribution of individuals with no pre-infection mirrored these trends, with Italian nurses and physicians forming most non-pre-infected cases, while Germany-Munich consistently showed minimal representations in both categories. These findings, as depicted in Figure 2, highlight pronounced regional and professional variations in pre-infection and no-pre-infection rates, with Italian healthcare professionals being disproportionately affected compared to their counterparts in other regions.

### 3.2. Exploring the Serological Response to Vaccination

#### 3.2.1. Unadjusted Response Assessment

The Bass model consistently surpassed linear and non-linear regression models across all vaccine dose intervals, as demonstrated by stable and positive coefficients of determination (Supplemental Table S2). Anti-S levels peaked at week 20 post first dose in non-pre-infected individuals, remaining above the threshold for 40 weeks, while pre-infected individuals exhibited a linear decline (Supplemental Figure S3). External factors sustained hybrid immunity in pre-infected individuals, whereas internal factors drove adaptive immunity in non-pre-infected individuals (Supplemental Table S3, Table 1). From Dose 2 to Dose 3, peak antibody levels were observed at week 30 for the non-pre-infected group and at week 20 for the pre-infected group, with the model accurately reflecting these temporal dynamics (Supplemental Figure S3). Prolonged above-threshold levels indicated that the internal factor was driving hybrid adaptive immunity in the pre-infected group between weeks 10 and 58 post second dose (Supplemental Table S3, Table 1, Supplemental Figure S3). In the interval from Dose 3 to Dose 4, peaks occurred at week 20 for non-pre-infected individuals, while no peak was detected in pre-infected individuals (Supplemental Figure S3). The Bass model demonstrated a robust fit for both groups (Supplemental Table S2), indicating hybrid adaptive immunity in the pre-infected group (Table 1). Following Dose 4, anti-S levels remained elevated, with the model accurately capturing the trends in both scenarios, reflecting adaptability in both groups (Table 1). Overall, the model confirms vaccine-induced immunity after the first dose and enhanced adaptive immunity in pre-infected cases following booster doses, underscoring the importance of long-term booster strategies to reach hybrid immunity.

#### 3.2.2. Adjusted Response Analysis

The study aims to propose a novel factor-based prediction of immune responses to COVID-19 among HCWs by systematically adjusting key immuno-epidemiological parameters based on factors such as gender, age, and occupational roles. These adjustments will be rigorously evaluated at critical immunological intervals between vaccine doses (first to second, second to third, third to fourth, and after the fourth dose). By incorporating prior infection status, this approach aims to provide definitive evidence of vaccine-induced, hybrid, or adaptive immunological memory, thereby facilitating a more precise assessment of vaccine effectiveness, especially in the absence of sufficient historical data and immunological cell dynamics.

In the analysis of immune response predictions between Dose 1 and Dose 2, the Bass model demonstrated superior performance compared to both linear and non-linear regression models in forecasting anti-S levels (Supplemental Table S2). Specifically, for male participants, the Bass model effectively captured variations in non-pre-infected (minimal predictive value) and pre-infected individuals, significantly outperforming linear and non-linear regression counterparts, which exhibited negative or low predictive values. Female participants showed a similar trend, with the Bass model providing a modest advantage over both regression approaches. Overall, the regression models across various age and occupational groups generally yielded lower forecasted fit values than those of the Bass model (Supplemental Table S2). The trajectory of anti-S levels revealed a concave downward pattern across most groups, except for women and individuals aged 30–39 years in pre-infection data. Notably, peak anti-S levels were observed at week 6 for non-pre-infected males and at week 13 for individuals aged 40–49 years (Supplemental Figures S3–S6). Furthermore, the External Dominance Ratio was significantly higher in pre-infected men (22.65) compared to non-pre-infected men (0.37), with a similar pattern observed in women (24.12 vs. 0.12). Results indicate a pronounced external dominance in pre-infected cases across most occupational roles, except for technicians (Supplemental Table S3). Throughout most of the interval, anti-S levels remained above the threshold, indicating a robust vaccine-induced immune response prior to the first booster. However, pre-infected cases, particularly among technicians and individuals aged 40–49, demonstrated lower levels at both the beginning and end of the interval, suggesting a less durable hybrid immune response. While both genders achieve hybrid immunity in pre-infected cases, non-pre-infected men demonstrated adaptive immunological memory for four months post-vaccination. In contrast, non-pre-infected women exhibited adaptive responses after the first month and maintained this adaptivity for approximately one year. Among age groups, only non-pre-infected individuals over 50 years of age exhibited a lack of adaptive memory following vaccination, in stark contrast to pre-infected cases, which achieved hybrid immunity. In terms of occupational roles, vaccine-induced immunity consistently provided adaptive memory for physicians, administrators, and other HCWs, regardless of prior infection history, while pre-infected technicians uniquely attained hybrid adaptive immunity, unlike their counterparts who achieved only hybrid immunity.

During the interval between Dose 2 and Dose 3, the Bass model continued to exhibit superior predictive capabilities compared to linear and non-linear regression models. For instance, for men without prior infection, the Bass model achieved a coefficient of determination that significantly exceeded those of both linear and non-linear regression models, which produced negative values. Similarly, for pre-infected women, the Bass model demonstrated a higher coefficient of determination than both regression models, indicating a superior predictive fit (Supplemental Table S2). Across various age groups and occupational roles, the Bass model consistently outperformed other models, maintaining positive predictive values. For instance, in individuals under 29 years with prior infection, the Bass model’s performance was approximately 20% better than that of linear regression. Specifically, in the nursing occupational group, the Bass model achieved a noteworthy predictive capability for non-pre-infected individuals, vastly exceeding the performance of both alternative regression models. Throughout this period, men typically exhibited a pronounced increase in antibody levels, peaking at week 22 for those without prior infection and week 30 for those with prior infection; these levels remained above the threshold for extended durations. The External Dominance Ratio for men, regardless of prior infection, revealed a strong influence of external factors, with those having prior infection exhibiting hybrid immunity. In contrast, females displayed more gradual patterns of antibody levels and maintained their levels above the threshold for a longer duration than their male counterparts; for instance, non-pre-infected cases demonstrated adaptive immunity. Age-related differences revealed that younger individuals maintained consistent antibody levels throughout the study period, while older individuals exhibited prolonged responses when pre-infected. In the context of occupational roles, administrative staff and pre-infected technicians exhibited early peaks in antibody levels due to strong external factor effects, while nurses and physicians experienced later peaks and more sustained antibody levels associated with prior infection, indicating hybrid immunity. Additionally, non-pre-infected cases among other HCWs achieved adaptive memory (Supplemental Figure S6).

During the interval between Dose 3 and Dose 4, the Bass model consistently outperformed traditional regression models in predicting anti-S levels across various demographics, including gender, age groups, and job titles. The model effectively captured anti-S trends, demonstrating a notable predictive advantage for non-pre-infected men and pre-infected women, with the latter showing a substantial improvement over linear regression (Supplemental Table S2). Men exhibited a concave downward pattern with peaks at week 15 for non-pre-infected individuals and at week 25 for pre-infected individuals, while women displayed a linear decline without clear peaks. Both sexes maintained anti-S levels above the threshold for at least one year, indicating sustained immune protection from vaccination. The External Dominance Ratio indicated stronger external factor effects in both genders. Across all age groups, the Bass model yielded better predictive fits compared to other models, with younger individuals peaking earlier and maintaining levels above the threshold throughout the study period. Occupationally, administrators, technicians, and nurses exhibited improved predictive model fits, with peaks observed at weeks 25 and 30 for pre-infected technicians and physicians, respectively (Supplemental Table S2 and Figure S6). Despite some evidence of immunological adaptivity in non-pre-infected physicians and other HCWs under the age of 39, stable adaptive immune memory was not universally established across all groups following the second booster (Table 1).

The predictive analysis of anti-S antibody kinetics following Dose 4 revealed less distinct patterns across gender, age groups, and job titles, influenced by previous infection status. Men exhibited stronger predictive capabilities in the Bass diffusion model compared to women, demonstrating a significantly better fit for non-pre-infected cases, with anti-S levels in men peaking at week 3 without prior infection and week 12 with prior infection, almost constantly remaining above the threshold throughout the predictive analysis period. In contrast, women displayed a lower model fit, characterized by a linear decline in the non-pre-infection group and a peak at week 18 for pre-infected cases, maintaining levels above the threshold from weeks 3 to 32. Age group analysis revealed considerable variability in anti-S kinetics, with individuals aged 30–39 years demonstrating the highest predictive model fit. This group peaked at week 7 for pre-infected cases, while non-pre-infected cases did not exhibit a peak, maintaining anti-S levels above the threshold throughout the study. Younger individuals (≤29 years) with no prior infection exhibited significant responses, peaking at week 5, while those aged 50 and older maintained levels above the threshold for varying durations depending on their pre-infection status. Job title adjustments indicated that administrative workers and nurses exhibited the most consistent and high predictive model fits among non-pre-infected cases, peaking at week 2. Physician exhibited lower predictive model fits in non-pre-infected groups but demonstrated stronger responses in pre-infected groups. Overall, the third booster effectively induced adaptive immunity across most demographic categories, despite variability in sustained hybrid immune memory among different groups.

### 3.3. Vaccine-Associated COVID-19 Occurrence

#### 3.3.1. Unadjusted COVID-19 Occurrence

Examining the entire cohort dataset, the probability of contracting COVID-19, based on Anti-N results and PCR tests, differs significantly between pre-infected and non-pre-infected cases across dose intervals. Between Dose 1 and Dose 2, pre-infected cases had a higher probability of occurrence (5%) compared to non-pre-infected cases (0.1%), with a strongly increasing trend over time (Supplemental Figure S7A). Between Dose 2 and Dose 3, the probability rose to 40% for non-pre-infected cases and 22% for pre-infected cases, with a notable upward trend in the pre-infected group over time (Supplemental Figure S7B). Between Dose 3 and Dose 4, the probability decreased to 11% for non-pre-infected and 21% for pre-infected cases, reflecting a downward trend for pre-infected individuals (Supplemental Figure S7C). Following Dose 4, the probability declined to 0.04% for non-pre-infected and 0.32% for pre-infected cases, showing a clear reduction in COVID-19 risk for pre-infected individuals (Supplemental Figure S7D). The extent of the reduction varied, with pre-infected cases showing a smaller percentage decrease than non-pre-infected cases across all intervals.

#### 3.3.2. Adjusted COVID-19 Occurrence by Demographics

The correlation between COVID-19 occurrence and pre-infection status, as determined by Anti-N results and PCR testing, demonstrates the effectiveness of the vaccine, which in turn highlights the impact of internal and external factors on achieving antibody thresholds in different demographic groups. In the first interval (Dose 1 to Dose 2), administrative staff and nurses had high antibody levels, resulting in a lower likelihood of occurrence, with protection lasting 50 weeks after the first dose. In the second interval (Dose 2 to Dose 3), immunity increased, especially among administrative staff, whose probability of occurrence decreased to 18%, while nurses showed improved protection, with a probability of 25%. Age and gender differences were observed, with younger individuals showing better protection than older groups, and women showing different antibody levels depending on their pre-infection status. In the third interval (Dose 3 to Dose 4), the probability of occurrence decreased to 24% for nurses, reflecting improved protection from the third dose, while technicians had a higher probability of 21%. By the fourth dose, the probability of occurrence dropped significantly in all groups, with administrative staff and nurses having probabilities of occurrence close to zero (0.2% and 0.3%, respectively), suggesting almost complete immunity. Physicians and older people had slightly higher probability, indicating lower anti-S levels. Overall, the data highlight the effectiveness of multiple doses of vaccine in boosting immunity and reducing the risk of infection in different demographic groups, particularly when anti-S levels are above protective thresholds (Supplemental Figures S7–S10).

## 4. Discussion

### 4.1. Antibody Kinetics and Immune Response

Our analysis highlights the robustness of the Bass diffusion model in clarifying patterns of antibody titer variation, temporal trends in both hybrid and adaptive memory formation, and vaccine effectiveness throughout the COVID-19 pandemic. The model demonstrates superior predictive capabilities compared to traditional regression models, thereby facilitating a nuanced understanding of the types of immune responses. By examining variations in antibody levels between vaccine doses and incorporating demographic factors and pre-infection status, we evaluate the robustness of the Bass model in detecting the development of hybrid immunity and adaptive memory. This analysis highlights the critical importance of distinguishing between the dominant influences of internal and external factors, as this distinction is essential for understanding whether immunity is primarily shaped by natural adaptation or vaccine-induced responses. Furthermore, analyzing variations in antibody titers relative to their immunological thresholds and identifying the internal and external factors that influence these variations enhances our understanding of immune responses, immunological memory formation, and overall vaccine effectiveness [33,34,35].

Between Dose 1 and Dose 2, the Bass diffusion model exhibited a robust fit, demonstrating superior predictive capabilities compared to traditional models. In non-pre-infected individuals, anti-S levels remained above the threshold for 40 weeks, while pre-infected individuals sustained these levels throughout the entire observation period. The model parameters indicated that antibody titers in non-pre-infected individuals were predominantly influenced by internal factors, whereas external factors played a more significant role in those with a history of infection. Generally, non-pre-infected individuals maintained adaptive immunity for up to 10 months, whereas pre-infected individuals displayed mostly hybrid immunity. Notably, men and women without prior infection developed adaptive immunity for at least five months, while those with prior infection failed to establish adaptive memory even after more than eight months. In non-pre-infected individuals under 49 years of age, adaptive immunity was preserved for up to one year. Among occupational groups, administrators, physicians, and other HCWs demonstrated adaptive immunity, while technicians and nurses did not exhibit this response even after one year. These findings align with those of [36], which discuss the stability of antibody titers and the durability of hybrid vaccine-induced immunological memory following prior infection. However, they challenge the conclusions of [37], which advocate for long-term immune protection resulting from prior infection.

Between Dose 2 and Dose 3, a pattern emerged that aligned with the findings of [38]: anti-S antibody levels remained above the threshold for periods ranging from a few months to just over a year in non-pre-infected individuals and up to one and a half years in pre-infected individuals, following a concave downward trend. However, pre-infected individuals appeared to be primarily influenced by external factors, while adaptive immunity was observed in non-pre-infected women under 39 years of age in other healthcare roles, consistent with the findings of [39]. Notably, long-term immunity developed in pre-infected individuals, aligning with the widespread development of hybrid immunity reported by [40], which occurred regardless of differences in demographic characteristics.

Between Dose 3 and Dose 4, anti-S antibody levels remained above the threshold for 15 months in individuals without prior infection, while those with prior infection maintained these levels throughout the observation period [41]. Notably, external factors were critical in influencing antibody persistence in the non-pre-infected group, whereas external factors also played a dominant role in sustaining elevated antibody levels in the previously infected group. Immunological adaptivity was observed in non-pre-infected technicians, physicians, and other healthcare workers, as well as in pre-infected technicians under the age of 39. However, demographic analysis indicated that immunity was predominantly vaccine-induced, with no evidence of a long-lasting adaptive immune response [42].

After Dose 4, pre-infected individuals exhibited a concave downward trajectory in anti-S levels, while non-pre-infected individuals experienced a linear decline. Internal factors consistently influenced both groups, except for pre-infected cases among nurses and physicians over the age of 50. Antibody levels in pre-infected individuals remained above the threshold for 1 to 10 months, whereas non-pre-infected individuals maintained levels above the threshold for the entire one-year study period [7,43]. The third booster elicited a broad and robust adaptive immune response across a wide range of demographic categories, regardless of pre-infection status. Overall, regardless of prior infection status, age-related patterns indicated stronger adaptive immunity in all age groups below 50 years of age.

However, individuals under 30 years of age who received only one dose of the vaccine were more likely to develop COVID-19. Gender differences were also observed, with female HCWs more likely to contract COVID-19 than their male counterparts, potentially due to higher factor risks. Occupational analysis revealed that administrative staff and physicians had the lowest likelihood of infection, reflecting the highest anti-S levels and vaccine effectiveness.

### 4.2. Study Limitations and Strengths

This study provides valuable insights while acknowledging notable limitations. Variability in serological data, including insufficient information on Anti-N tests and differing testing methods across cohorts, may introduce bias. Additionally, the predominance of Italian sites may limit the generalizability of findings across broader European settings. Furthermore, the assumptions of the Bass diffusion model could oversimplify the complexities of immune responses, limiting its applicability to diverse populations. Demographic variability and unmeasured confounders may also affect results, while external factors such as hybrid vaccination strategies require further exploration.

Notably, the study’s strengths lie in its innovative methodology for assessing immunity amid limited data on immune cell dynamics and its focus on antibody fluctuations. The large sample size, rigorous methodology, and comprehensive analysis of factors such as pre-infection status significantly enhance our understanding of immune responses and vaccine effectiveness. Future studies should build upon these findings by incorporating additional data on Anti-N tests and immune cell activation, thereby refining immunity predictions and further elucidating the complexities of immune responses in vaccinated populations in alignment with our results.

## 5. Conclusions

This study presents a novel methodology for elucidating the immune response to COVID-19 vaccination through the application of the Bass diffusion model, which systematically anticipates antibody levels and their generators. The findings indicate that individuals with prior infection exhibit significantly higher and more sustained antibody levels than those without prior infection. Anticipating the effect of internal factors, particularly immune cell activation rates, is crucial for maintaining elevated antibody levels over time, while the anticipated effect of external factors, such as subsequent vaccinations or infections, primarily rearranges immune responses in non-pre-infected individuals. This underscores the critical importance of accounting for prior infection status when evaluating vaccine effectiveness and the duration of protection. The study further confirms that antibody levels in non-pre-infected individuals decline following each vaccine dose, while those with prior infection sustain elevated levels for a more extended period. Overall, the Bass model effectively captures these complex dynamics, offering valuable insights into the formation of immune types following vaccination. These insights can contribute to the optimization of vaccination strategies and the formulation of public health policies.

## Figures and Tables

**Table 1:** Occurrence of immunity types post vaccine doses (dose 1 to dose 4), stratified by pre-infection status, demographic characteristics, and job titles: This table summarizes the dominant factor types (internal or external) and their resulting immunity types (vaccine-induced, hybrid, adaptive, or hybrid adaptive) across dose intervals among vaccinated healthcare workers. The immunity types are color-coded as follows: light green for vaccine-induced, light purple for hybrid, peach for adaptive, and dark purple for hybrid adaptive.

		Between Dose 1–Dose 2 (N = 2138)				Between Dose 2–Dose 3 (N = 28,426)			
		Non-Pre-Infected		Pre-Infected		Non-Pre-Infected		Pre-Infected	
		Effects Predominant	Immunity Type	Effects Predominant	Immunity Type	Effects Predominant	Immunity Type	Effects Predominant	Immunity Type
	**Unadjusted**	Internal Factors	Adaptive (N = 54)	External Factors	Hybrid (N = 1011)	External Factors	Vaccine Induced (N = 8214)	Internal Factors	Hybrid Adaptive (N = 5332)
**Gender**	**male**	Internal Factors	Adaptive (N = 15)	External Factors	Hybrid (N = 260)	External Factors	Vaccine Induced (N = 2132)	External Factors	Hybrid (N = 1411)
	**female**	Internal Factors	Adaptive (N = 39)	External Factors	Hybrid (N = 751)	Internal Factors	Adaptive (N = 6082)	External Factors	Hybrid (N = 3921)
**Age-group**	**age ≤ 29**	Internal Factors	Adaptive (N = 6)	External Factors	Hybrid (N = 113)	External Factors	Vaccine Induced (N = 1061)	External Factors	Hybrid (N = 807)
	**30 ≤ age ≤ 39**	Internal Factors	Adaptive (N = 9)	External Factors	Hybrid (N = 161)	Internal Factors	Adaptive (N = 1586)	External Factors	Hybrid (N = 1025)
	**40 ≤ age ≤ 49**	Internal Factors	Adaptive (N = 15)	External Factors	Hybrid (N = 292)	External Factors	Vaccine Induced (N = 2052)	External Factors	Hybrid (N = 1393)
	**50 ≤ age**	External Factors	Vaccine Induced (N = 24)	External Factors	Hybrid (N = 445)	External Factors	Vaccine Induced (N = 3514)	External Factors	Hybrid (N = 2107)
**Job title**	**administration**	Internal Factors	Adaptive (N = 6)	External Factors	Hybrid (N = 85)	External Factors	Vaccine Induced (N = 1022)	External Factors	Hybrid (N = 452)
	**technician**	Not Detected	Not Detected	Internal Factors	Hybrid Adaptive (N = 70)	Not Detected	Not Detected	External Factors	Hybrid (N = 384)
	**nurse**	External Factors	Vaccine Induced (N = 12)	External Factors	Hybrid (N = 434)	External Factors	Vaccine Induced (N = 2698)	External Factors	Hybrid (N = 2253)
	**physician**	Internal Factors	Adaptive (N = 14)	External Factors	Hybrid (N = 166)	External Factors	Vaccine Induced (N = 2002)	External Factors	Hybrid (N = 1101)
	**other HCWs**	Internal Factors	Adaptive (N = 15)	External Factors	Hybrid (N = 195)	Internal Factors	Adaptive (N = 1498)	External Factors	Hybrid (N = 1063)
		**Between Dose 3–Dose 4** **(N = 12,222)**				**After Dose 4** **(N = 128)**			
		**Non-Pre-Infected**		**Pre-Infected**		**Non-Pre-Infected**		**Pre-Infected**	
		**Effects Predominant**	**Immunity Type**	**Effects Predominant**	**Immunity Type**	**Effects Predominant**	**Immunity Type**	**Effects Predominant**	**Immunity Type**
	**Unadjusted**	External Factors	Vaccine Induced (N = 3312)	Internal Factors	Hybrid Adaptive (N = 5232)	Internal Factors	Adaptive (N = 11)	Internal Factors	Hybrid Adaptive (N = 67)
**Gender**	**male**	External Factors	Vaccine Induced (N = 822)	External Factors	Hybrid (N = 1278)	Internal Factors	Adaptive (N = 2)	Internal Factors	Hybrid Adaptive (N = 29)
	**female**	External Factors	Vaccine Induced (N = 2490)	External Factors	Hybrid (N = 3954)	Internal Factors	Adaptive (N = 9)	Internal Factors	Hybrid Adaptive (N = 38)
**Age-group**	**age ≤ 29**	Internal Factors	Adaptive (N = 403)	External Factors	Hybrid (N = 610)	Not Detected	Not Detected	Internal Factors	Hybrid Adaptive (N = 4)
	**30 ≤ age ≤ 39**	Internal Factors	Adaptive (N = 541)	External Factors	Hybrid (N = 1000)	Internal Factors	Adaptive (N = 2)	Internal Factors	Hybrid Adaptive (N = 9)
	**40 ≤ age ≤ 49**	External Factors	Vaccine Induced (N = 855)	External Factors	Hybrid (N = 1410)	Internal Factors	Adaptive (N = 3)	Internal Factors	Hybrid Adaptive (N = 10)
	**50 ≤ age**	External Factors	Vaccine Induced (N = 1513)	External Factors	Hybrid (N = 2211)	Internal Factors	Adaptive (N = 6)	External Factors	Hybrid (N = 44)
**Job title**	**administration**	External Factors	Vaccine Induced (N = 424)	External Factors	Hybrid (N = 471)	Internal Factors	Adaptive (N = 1)	Internal Factors	Hybrid Adaptive (N = 4)
	**technician**	Internal Factors	Adaptive (N = 256)	Internal Factors	Hybrid Adaptive (N = 517)	Not Detected	Not Detected	Internal Factors	Hybrid Adaptive (N = 7)
	**nurse**	External Factors	Vaccine Induced (N = 1043)	External Factors	Hybrid (N = 2202)	Internal Factors	Adaptive (N = 1)	External Factors	Hybrid (N = 22)
	**physician**	Internal Factors	Adaptive (N = 636)	External Factors	Hybrid (N = 937)	Internal Factors	Adaptive (N = 2)	External Factors	Hybrid (N = 22)
	**other HCWs**	Internal Factors	Adaptive (N = 532)	External Factors	Hybrid (N = 950)	Not Detected	Not Detected	Internal Factors	Hybrid Adaptive (N = 7)

## Data Availability

Raw data supporting the conclusions of this article are available on reasonable request from the Principal Investigators of the participating cohorts.

## Supplementary Materials

The following supporting information can be downloaded at: https://www.mdpi.com/article/10.3390/vaccines13060611/s1, Figure S1: Population Map; Figure S2: Population Study; Figure S3: Bass diffusion model of antibody titers by pre-infection status; Figure S4: Bass diffusion analysis of weekly average Anti-S antibody titers stratified by gender and pre-infection status; Figure S5: Bass Diffusion Analysis of Weekly Average Antibody Titers Stratified by Age Group and Pre-Infection Status; Figure S6: Bass Diffusion Analysis of Weekly Average Antibody Titers by Job Title and Pre-Infection Status; Figure S7: Probability of COVID-19 occurrence; Figure S8: Probability of covid-19 occurrence by gender and pre-infection status; Figure S9: Probability of covid-19 occurrence by age group and pre-infection status; Figure S10: Probability of covid-19 occurrence by job title and pre-infection status; Table S1: SARS-CoV-2 antibody measurement methods and cohort-specific mean serology results; Table S2: Model fit evaluation; Table S3: Predominance Exposure Effects.
