# Supplementary material for: Antibody Kinetics of Immunological Memory in SARS-CoV-2-Vaccinated Healthcare Workers—The ORCHESTRA Project
Source: Vaccines (Basel). 2025 Jun 5;13(6):611. doi: 10.3390/vaccines13060611 (PMC12197645; doi:10.3390/vaccines13060611)
Supplement: Supplementary file 1 [file vaccines-13-00611-s001.zip › Supplementary Figures.pdf]

Supplementary figures for "Antibody Kinetics of Immunological Memory in SARS-CoV-2 Vaccinated Healthcare Workers – ORCHESTRA Project."

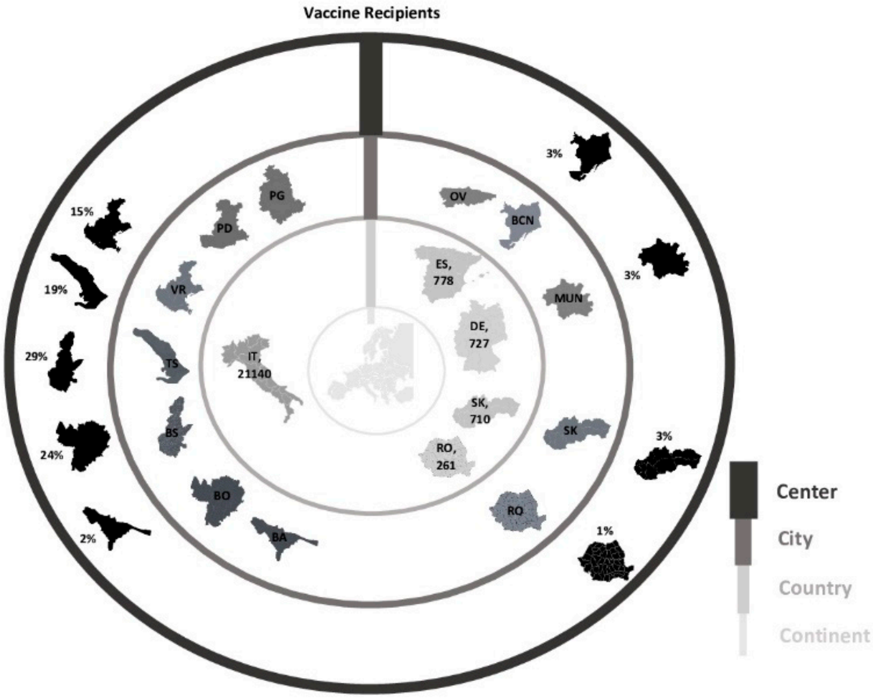

Figure S1. Population Map. Nine of the eleven cohorts of vaccinated HCWs in the ORCHESTRA project were selected to evaluate immune response patterns.

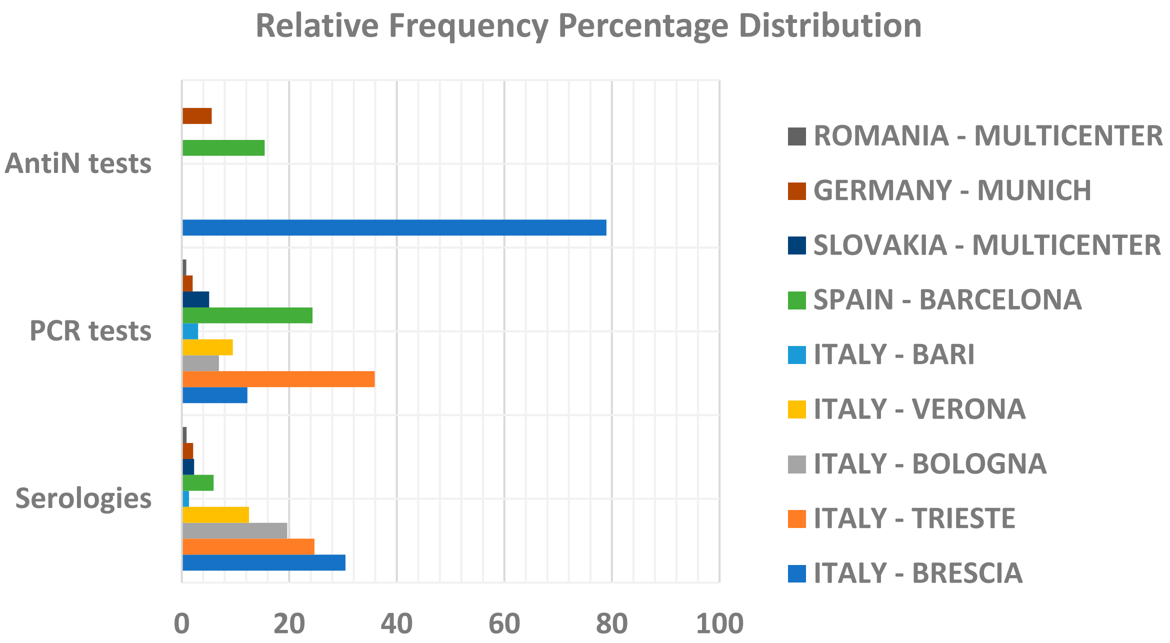

Figure S2. Population Study: Data from nine major cohorts, mainly Italian, were used to create a predictor of immune system behaviour in response to coronavirus, focusing on anti-S and anti-N antibody levels with the corresponding PCR tests.

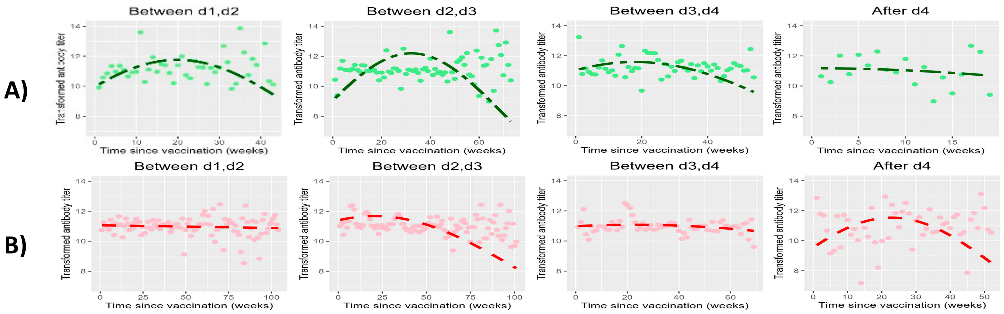

Figure S3. Bass diffusion model of antibody titers by pre-infection status: The figure elucidates the weekly average Anti-S antibody titers following vaccination and associated boosters, as predicted by the Bass Diffusion model and meticulously adjusted for pre-infection status. Panel (**A**) delineates the diffusion trends for individuals devoid of prior infection, capturing the dynamics between vaccine doses, while Panel (**B**) concentrates on those with a history of infection. The green dashed lines signify predictions for non-pre-infected individuals, whereas the red solid lines denote predictions for previously infected cases, thereby accentuating the disparities in antibody response kinetics between these two groups.

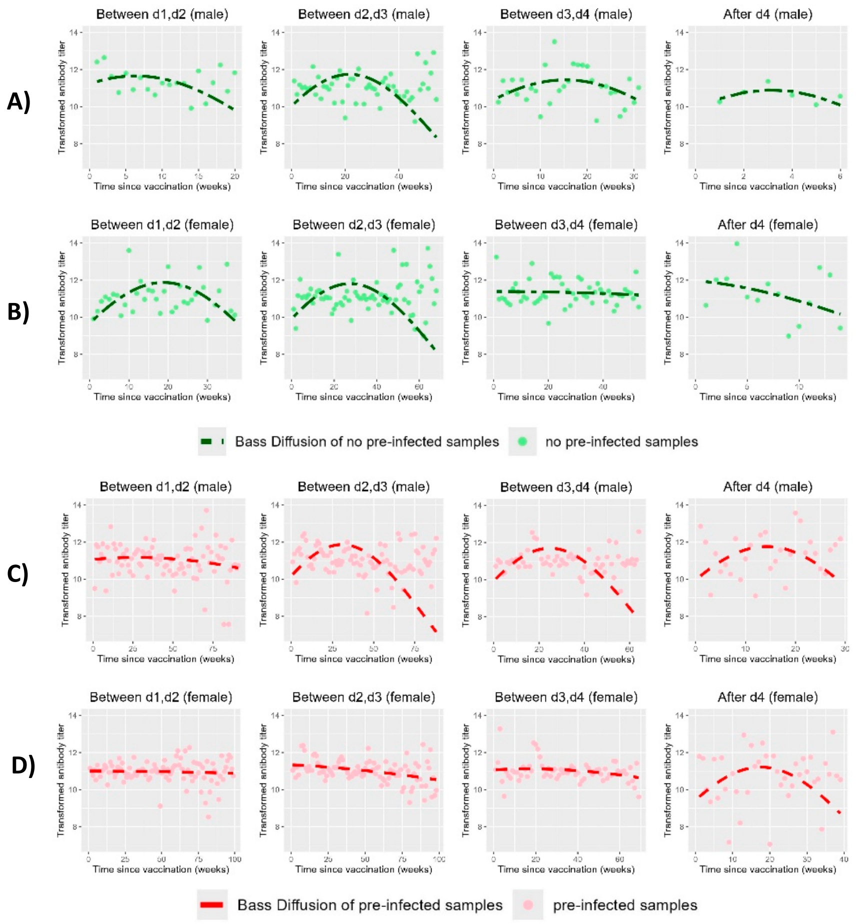

Figure S4. Bass diffusion analysis of weekly average Anti-S antibody titers stratified by gender and pre-infection status: The figure provides a comprehensive prediction of weekly average Anti-S antibody titers following the 1st, 2nd, 3rd, and 4th vaccine doses, utilizing the Bass Diffusion model and adjusted for gender in conjunction with pre-infection status. It is organized into four panels: Panels (**A**) and (**B**) illustrate the Anti-S trends for non-pre-infected men and women, respectively, while Panels (**C**) and (**D**) present the corresponding trends for pre-infected men and women. The green dashed lines represent the predicted diffusion of antibody titers for non-pre-infected individuals, whereas the red solid lines indicate predictions for those with prior infection. This comparative analysis underscores the differential kinetics of antibody responses influenced by both gender and pre-infection status, aligning with observed trends related to external and internal exposure effects across various groups.

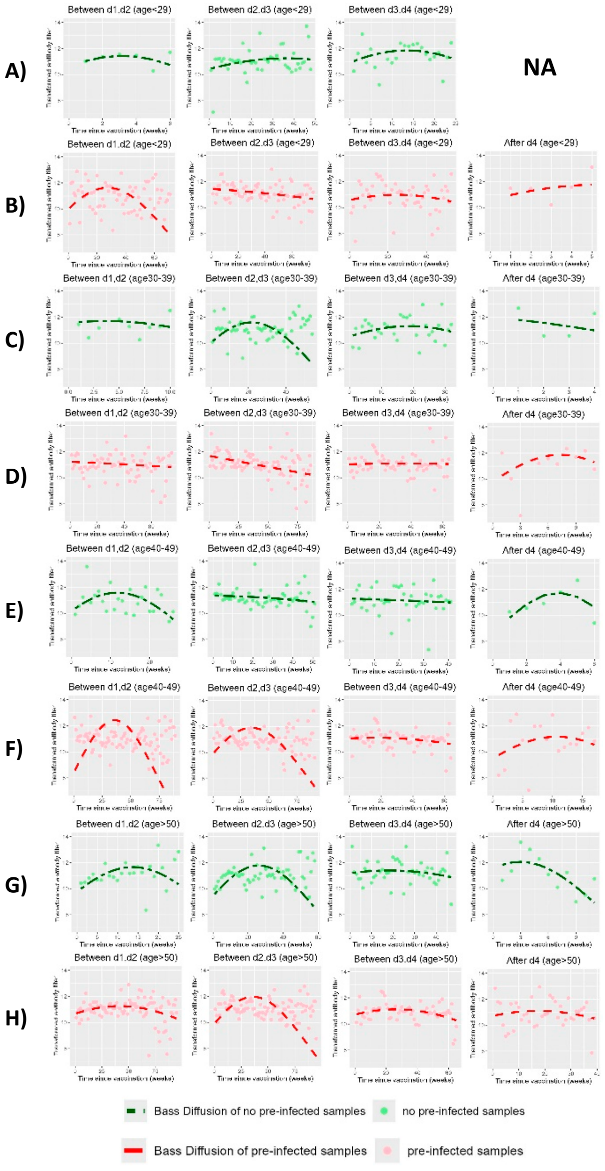

Figure S5. Bass Diffusion Analysis of Weekly Average Antibody Titers Stratified by Age Group and Pre-Infection Status: The figure presents a comprehensive prediction of weekly average Anti-S antibody titers following the 1st, 2nd, 3rd, and 4th vaccine doses, utilizing the Bass Diffusion model and adjusted for age groups and pre-infection status. It is structured into eight panels: Panels (**A**) and (**B**) illustrate predicted diffusion trends for non-pre-infected and pre-infected individuals under age 29, respectively; Panels (**C**) and (**D**) depict similar trends for the 30-39 age group; Panels (**E**) and (**F**) correspond to the 40-49 age group; and Panels (**G**) and (**H**) display predicted trends for individuals over age 50. The green dashed lines represent the predicted diffusion trends of antibody titers for non-pre-infected individuals, while the red solid lines denote those with prior infection. This stratified analysis emphasizes

the significant influence of age and prior infection on antibody kinetics, aligning with observed variations in external and internal exposure effects across different demographic groups.

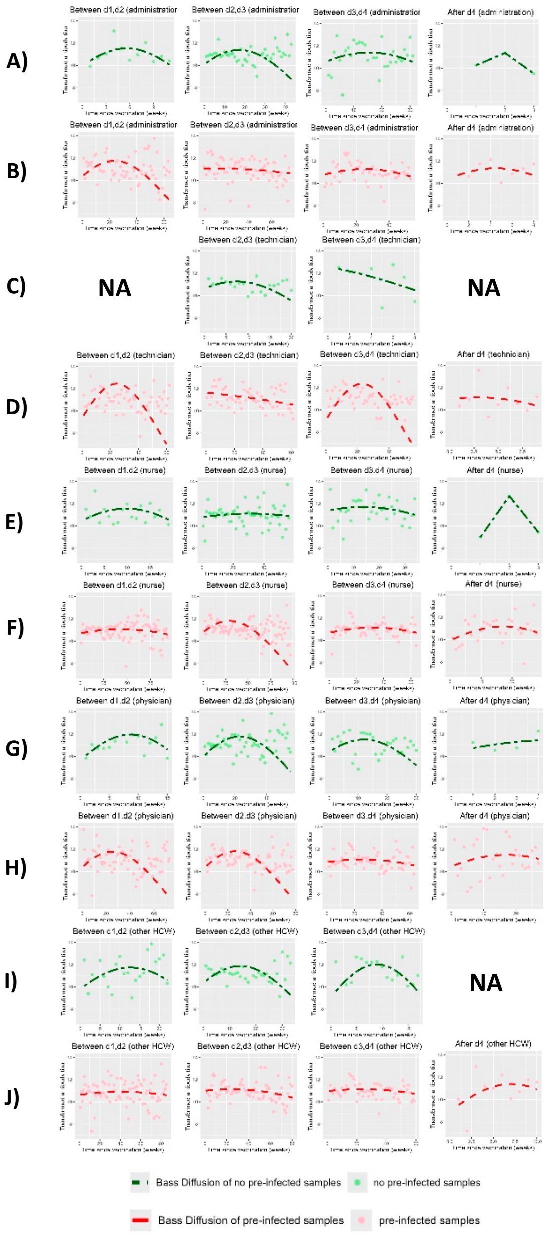

Figure S6. Bass Diffusion Analysis of Weekly Average Antibody Titers by Job Title and Pre-Infection Status: The figure depicts the Bass Diffusion model prediction of weekly average Anti-S antibody titers following the 1st, 2nd, 3rd, and 4th vaccine doses, adjusted for job titles and pre-infection status. It comprises eight panels: Panels (**A**) and (**B**) illustrate predicted diffusion trends for non-pre-infected and pre-infected individuals in administrative roles; Panels (**C**) and (**D**) present similar trends for technicians; Panels (**E**) and (**F**) correspond to nurses; and Panels (**G**) and

(**H**) display predicted trends for physicians and other healthcare workers. Additionally, Panels (**I**) and (**J**) provide predicted trends for other healthcare workers. The green dashed lines indicate the predicted diffusion of Anti-S antibody titers for non-pre-infected individuals, while the red solid lines represent those with prior infection. This analysis underscores the influence of job roles and prior infection on antibody kinetics, reflecting variations in exposure effects across different occupational groups.

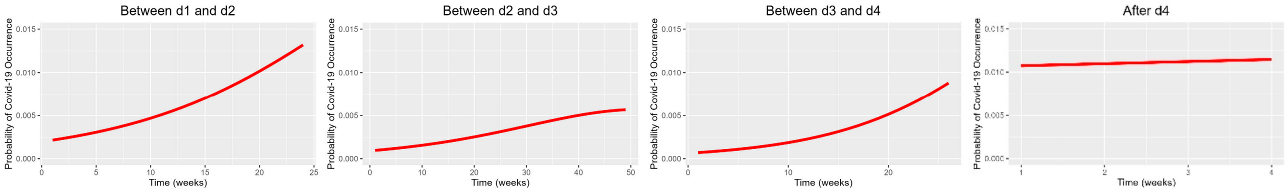

Figure S7. Probability of COVID-19 occurrence: This figure illustrates the probability of COVID-19 occurrence based on anti-N and PCR test results, stratified by pre-infection status. The red lines show trends in COVID-19 probability across vaccine doses for pre-infected cases, emphasizing the differential risk within the pre-infected and non-pre-infected cases group.

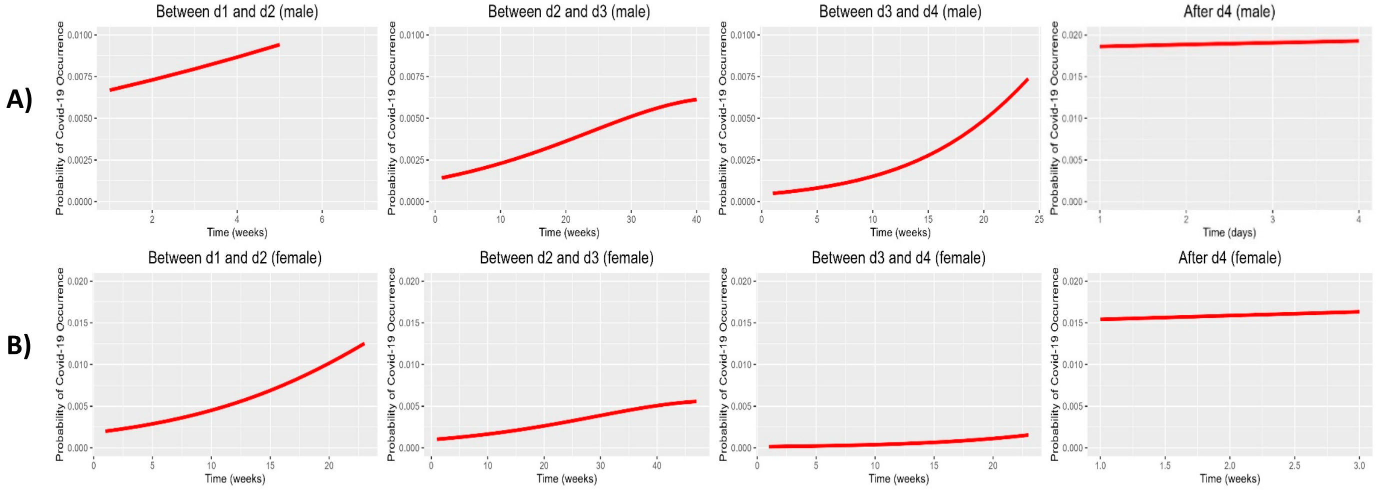

Figure S8. Probability of covid-19 occurrence by gender and pre-infection status: This figure illustrates the probability of COVID-19 occurrence based on anti-N and PCR test results, separated by gender and pre-infection

status. Panels (**A**) and (**B**) display this probability for pre-infected males and females, respectively. The red lines indicate the trend of COVID-19 probability between vaccine doses for each gender group. This visualization highlights gender-specific risk variations in COVID-19 occurrence in the population fraction between pre-infected and non-pre-infected cases.

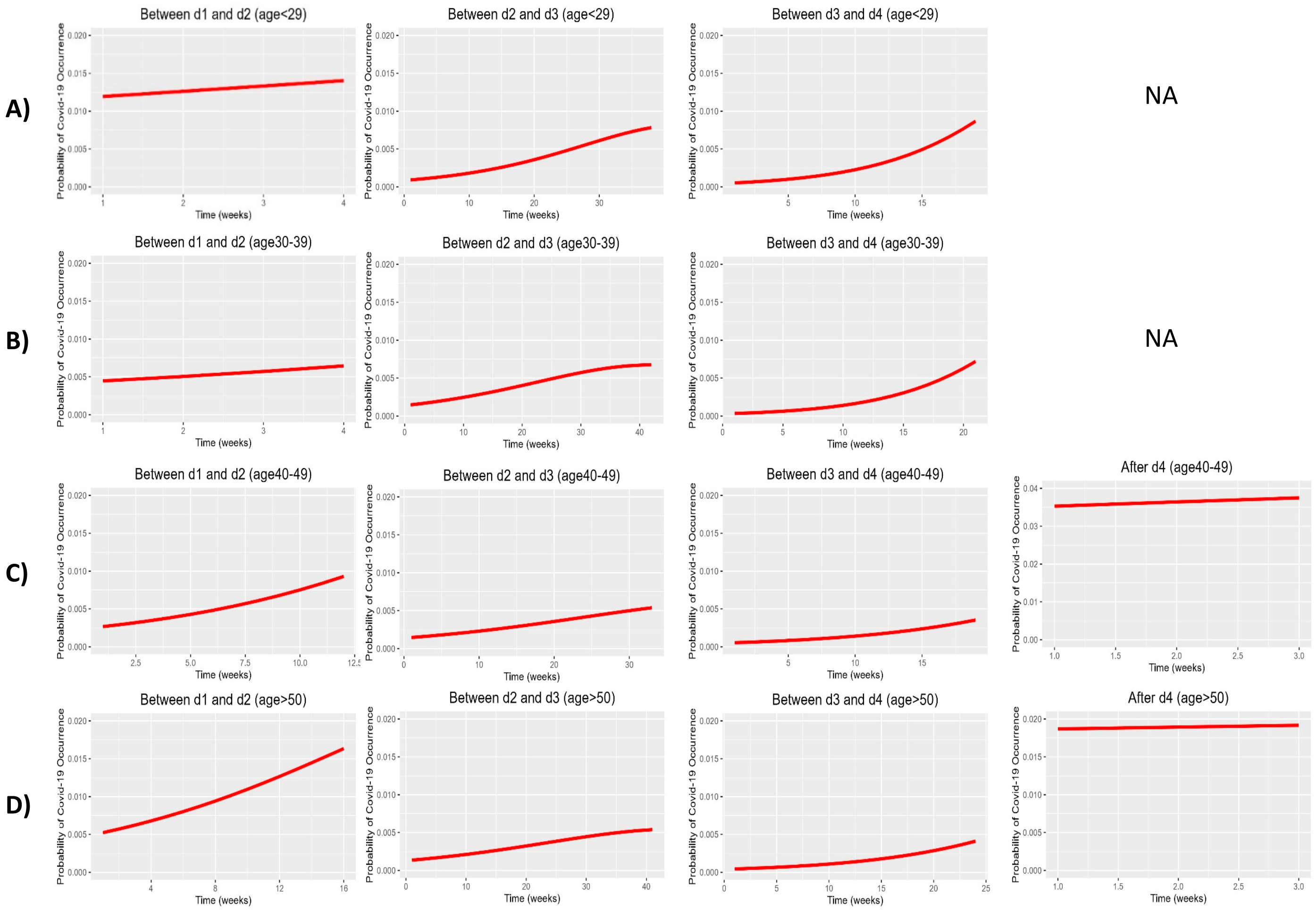

Figure S9. Probability of covid-19 occurrence by age group and pre-infection status: The figure shows the probability of COVID-19 occurrence by anti-N and PCR test statuses, categorized by age groups and pre-infection status. Panel (**A**) illustrates this probability for pre-infected individuals under 29 years old. Panel (**B**) shows results for those aged 30-39, panel (**C**) for ages 40-49, and panel (**D**) for individuals over 50. Red lines trace the probability trends between vaccine doses for each age group. This analysis underscores how age influences COVID-19 risk in the population fraction between pre-infected and non-pre-infected cases.

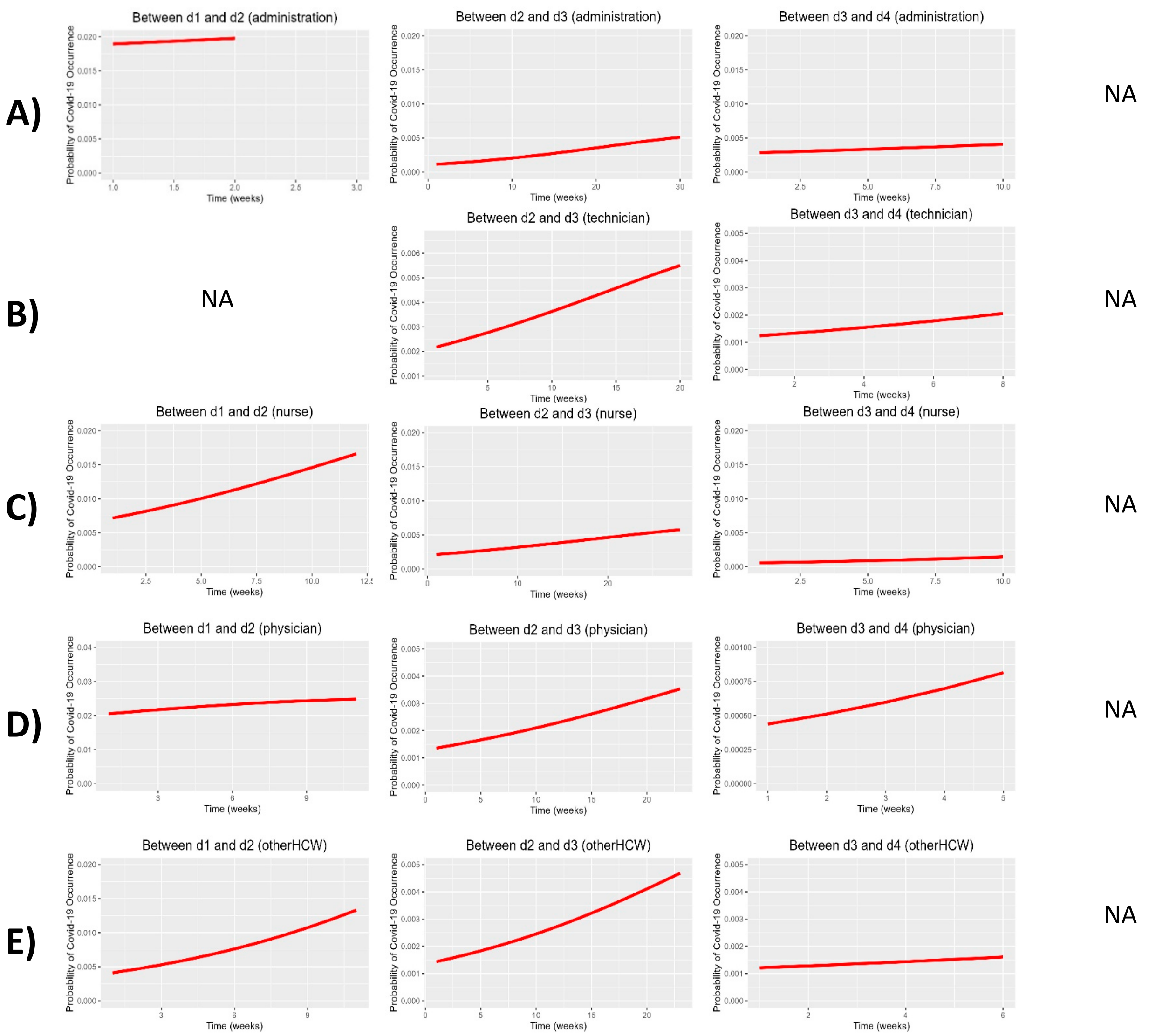

Figure S10. Probability of covid-19 occurrence by job title and pre-infection status: This figure depicts the probability of COVID-19 occurrence based on anti-N and PCR test results, segmented by job title and pre-infection status. Panels (**A**) shows the probability for pre-infected administrative staff, (**B**) for technicians, (**C**) for nurses, (**D**) for physicians, and (**E**) for other healthcare workers. The red lines illustrate the trend of COVID-19 probability between vaccine doses for each job title group. The results emphasize the influence of job roles on COVID-19 risk in the proportion of pre-infected and non-infected samples.
